# Striatal vessels receive phosphorylated tyrosine hydroxylase-rich innervation from midbrain dopaminergic neurons

**DOI:** 10.3389/fnana.2014.00084

**Published:** 2014-08-26

**Authors:** Domingo Afonso-Oramas, Ignacio Cruz-Muros, Javier Castro-Hernández, Josmar Salas-Hernández, Pedro Barroso-Chinea, Sonia García-Hernández, José L. Lanciego, Tomás González-Hernández

**Affiliations:** ^1^Department of Anatomy, Faculty of Medicine, University of La LagunaLa Laguna, Tenerife, Spain; ^2^Biomedical Technologies Institute (ITB, CIBICAN)La Laguna, Tenerife, Spain; ^3^Spanish Network of Neurodegenerative Diseases (CIBERNED)Madrid, Spain; ^4^Department of Pathology, University Hospital of Canary IslandsTenerife, Spain; ^5^Center for Applied Medical Research (CIMA), University of NavarraPamplona, Spain

**Keywords:** dopamine, midbrain, striatum, Ser 19, Ser 40, cerebral blood flow, Parkinson’s disease

## Abstract

Nowadays it is assumed that besides its roles in neuronal processing, dopamine (DA) is also involved in the regulation of cerebral blood flow. However, studies on the hemodynamic actions of DA have been mainly focused on the cerebral cortex, but the possibility that vessels in deeper brain structures receive dopaminergic axons and the origin of these axons have not been investigated. Bearing in mind the evidence of changes in the blood flow of basal ganglia in Parkinson’s disease (PD), and the pivotal role of the dopaminergic mesostriatal pathway in the pathophysiology of this disease, here we studied whether striatal vessels receive inputs from midbrain dopaminergic neurons. The injection of an anterograde neuronal tracer in combination with immunohistochemistry for dopaminergic, vascular and astroglial markers, and dopaminergic lesions, revealed that midbrain dopaminergic axons are in close apposition to striatal vessels and perivascular astrocytes. These axons form dense perivascular plexuses restricted to striatal regions in rats and monkeys. Interestingly, they are intensely immunoreactive for tyrosine hydroxylase (TH) phosphorylated at Ser19 and Ser40 residues. The presence of phosphorylated TH in vessel terminals indicates they are probably the main source of basal TH activity in the striatum, and that after activation of midbrain dopaminergic neurons, DA release onto vessels precedes that onto neurons. Furthermore, the relative weight of this “vascular component” within the mesostriatal pathway suggests that it plays a relevant role in the pathophysiology of PD.

## Introduction

DA is a neurotransmitter and neuromodulator involved in a wide range of brain functions including control of voluntary movements, reward-seeking behavior, cognitive processes and circuit formation during development (Schultz, 2007; Money and Stanwood, 2013; Morita et al., 2013). DA also exerts important actions in peripheral organs, with those being on the vascular dynamics control of particular relevance (Tayebati et al., 2011). Studies carried out in the 1970s and 1980s showed that DA and DA agonists have vasomotor effects on major extracerebral arteries and pial arterioles of the cortical surface, promoting a general increase in cerebral blood flow (Toda, 1976; Ingvar et al., 1983; Edvinsson et al., 1985). More recently, it has been shown that DA analogs also modify blood flow into discrete brain regions (Breiter et al., 1997; Marota et al., 2000), and that this effect is prevented by dopaminergic lesion (Chen et al., 1997; Nguyen et al., 2000; Jenkins et al., 2004). In sum, the involvement of DA in the regulation of regional cerebral blood flow is well-established nowadays. However, our knowledge on this issue is still fragmentary and relevant aspects have not been elucidated. For example, while some data suggest that vascular effects of DA require activation of postsynaptic D_1_ DA receptors (Knutson and Gibbs, 2007), dopaminergic contacts have also been described in small vessels of the monkey frontal cortex (Krimer et al., 1998), and DA receptor expression has been found in cultured endothelial cells and astrocytes (Bacic et al., 1991; Bal et al., 1994; Zanassi et al., 1999; Choi et al., 2006), suggesting a direct DA effect on vessels and surrounding astrocytes. On the other hand, most interest in cerebral blood flow regulation has been focussed on the cerebral cortex, whereas mechanisms operating in deep brain centers, including the striatum, have been less investigated. It is noteworthy that in spite of the striatum being the main target of midbrain dopaminergic inputs, there is no evidence that striatal vessels and surrounding astrocytes receive midbrain dopaminergic contacts. The clarification of this question could contribute to a better understanding of metabolic and hemodynamic events associated with striatal processing and their role in the pathophysiology of Parkinson’s disease (PD). This possibility has been explored here by using nigral injections of an anterograde tracer in combination with immunofluorescence for dopaminergic, vascular and astroglial markers, confocal microspcopy and dopaminergic lesion.

## Material and methods

Experiments were carried out on 16 male Sprague-Dawley rats (250–300 g; Charles River, L’Arbresle, France) and three male rhesus monkeys (*Macaca fascicularis*, 6–7 years old, 3.5–4.8 Kg). Nine rats were used for the injection of the anterograde neuronal tracer biotinylated dextran amine (BDA) in the dopaminergic cell groups of ventral midbrain, and seven rats for 6-hydroxydopamine (6-OHDA) lesion. The three monkeys were processed for immunofluorescence. Experimental protocols were approved by the Ethical committee of the University of La Laguna (Reference # 091/010), and are in accordance with the European Communities Council Directive of 22 September 2010 (2010/63/EU) regarding the care and use of animals for experimental procedures.

### Surgical procedures

BDA injections were performed according to Lanciego and Wouterlood (2006). A 10% solution of BDA (biotin, dextran 10 kDa; Molecular Probes, Leiden, The Netherlands) in 10 mM phosphate buffer pH 7.25 was iontophoretically delivered in the midbrain dopaminergic formation (1.2–1.8 mm lateral to midline, 5.7–6.0 mm posterior to bregma and 8.0–8.5 mm below the dura, according to Paxinos and Watson, 1998) using a glass micropipette (inner tip diameter 20–30 μm) and a positive-pulsed direct current (7 s on/off) for 7 min. The micropipette was left in place for 5 min before removal. Animals were killed 1 week after injection.

A rat model based on the intracerebroventricular injection of 6-OHDA was used for dopaminergic lesion. This model causes bilateral degeneration of mesostriatal dopaminergic neurons, and a motor syndrome composed of hypokinesia, purposeless chewing and catalepsy (Rodríguez et al., 2001; Rodríguez-Díaz et al., 2001; González-Hernández et al., 2004). Rats were injected in the third ventricle (midline, 2 mm posterior to bregma and 8 mm below the dura, according to Paxinos and Watson, 1998) with vehicle (0.9% saline solution with 0.3 μg/μl ascorbic acid, sham group, *n* = 3) or a single dose (400 μg) of 6-OHDA (6-hydroxydopamine hydrochloride, Sigma, St. Luis, MO; in 8 μl of vehicle per injection; 1 μl/min, 6-OHDA groups, *n* = 4). Anesthesia, pre-surgery treatment and intraventricular injection protocols followed Rodríguez et al. (2001). Bearing in mind that the bilateral degeneration of DA-cells can cause adipsia and aphagia (Zigmond and Stricker, 1973), the intake of food and water was monitored following the 6-OHDA injection. No body weight loss was observed and rats were killed 2 weeks after injection.

### Tissue processing

Animals were deeply anesthetized with an overdose of sodium pentobarbital and transcardially perfused with heparinized ice-cold 0.9% saline (150 ml in rats, 1 l in monkeys) followed by 4% paraformaldehyde in phosphate buffer saline 0.1 M pH 7.4 PBS; 300 ml in rats and 2.5 l in monkeys). The brains were then removed, the midbrain and forebrain blocks were stored in the same fixative at 4°C (8 h in rats and 18 h in monkeys), cryoprotected in a graded series of sucrose-PBS solutions and stored at −80°C until processing. Coronal sections (25 μm in rats, 40 μm in monkeys) were obtained with a freezing microtome, collected in parallel series and processed for single and double immunohistochemical labeling.

For detecting BDA-stained fibers, floating sections were immersed for 30 min in 3% H_2_O_2_ to inactivate endogenous peroxidase, washed several times in PBS, and then incubated for 90 min in either ExtrAvidin-peroxidase (1:5000, Sigma) or Cy2-conjugated ExtrAvidin (1:1000; Amersham, Buckinghamshire, England) and 0.3% TX-100 in PBS. In sections incubated in ExtrAvidin-peroxidase, stained fibers were visible after immersion for 5–10 min in 0.005% 3′-3′-diamiobenzidine tetrahydrochloride (DAB, Sigma) and 0.001% H_2_O_2_ in cacodylate buffer 0.5 N, pH 7.6.

Sections incubated in Cy2-conjugated ExtrAvidin were washed several times in PBS, and incubated for 60 min at room temperature (RT) in 4% normal goat serum (NGS, Jackson ImmunoResearch, West Grove, PA) in PBS, and overnight in PBS containing 2% NGS and one of the primary antibodies: mouse anti-tyrosine hyroxylase (TH) monoclonal antibody (Sigma, 1:12,000), rabbit anti-TH phosphorylated at Ser19 (THp19) polyclonal antibody (PhosphoSolutions, Aurora, CO, 1:2000), rabbit anti-THp31 polyclonal antibody (PhosphoSolutions, 1:600), rabbit anti-THp40 polyclonal antibody (PhosphoSolutions, 1:600), goat anti-dopamine transporter (DAT) polyclonal antibody (1:1000, Santa Cruz Biotechnology, Santa Cruz, CA), mouse anti-endothelial nitric oxide synthase (eNOS) monoclonal antibody (1:1000, Sigma), mouse anti-glial fibrilary acidic protein (GFAP) monoclonal antibody (1:2000, Sigma), or mouse anti-vimentin monoclonal antibody (1:400, Abcam, Cambrige, UK). Immunofluorescent labeling was visible after incubation for 3 h in Alexa Fluor 488-conjugated donkey anti-rabbit IgG (1:150; Molecular Probes, OR) and Rhodamine (TRITC) -conjugated goat anti-guinea-pig IgG (1:100; Jackson ImmunoResearch), Lissamine Rhodamine-conjugated donkey anti-goat IgG (1:100; Jackson ImmunoResearch) or Lissamine Rhodamine-conjugated goat anti-mouse IgG (1:100; Jackson ImmunoResearch) in PBS containing 1:200 NGS.

After several rinses, the sections were mounted on gelatinized slides, air dried, coverslipped with Vectashield (Vector), and examined under a confocal laser scanning microscopy system (Olympus FV1000, Hamburg, Germany) using appropriate filters. Sections were first examined using low-magnification lenses, and the areas of interest were analyzed at high magnification (×63 oil-immersion Plan-Apochromat objective lens, NA 1.4) at a resolution of 1024 × 1024 pixels, and acquired in Z-stack mode (10 μm total thickness, 6 z-steps). Selected high-resolution confocal images were then deconvoluted by using the MetaMorph 6.1r 0 software (Molecular Devices, Downingtown, PA). Deconvolution is a post-adquisition computational process for correcting the optical blur inherent to any image adquisition system, restoring the appearance of original image with the highest degree of confidence (Sibarita, 2005; Salin et al., 2008).

In addition, a quantitative analysis of double labeled terminals (THp19-DAT and THp40-DAT) was performed in high magnification images. Four striatal sections 100 μm apart were randomly selected in five rats. Four vessel-centered 200 μm × 200 μm square regions were also randomly selected in each section. Simple and double labeled terminals were counted within 100 μm from the putative border of the vessel. Data are expressed as the percentage of double labeled terminals per section ± standard error of the mean.

## Results

**Figure 1:**
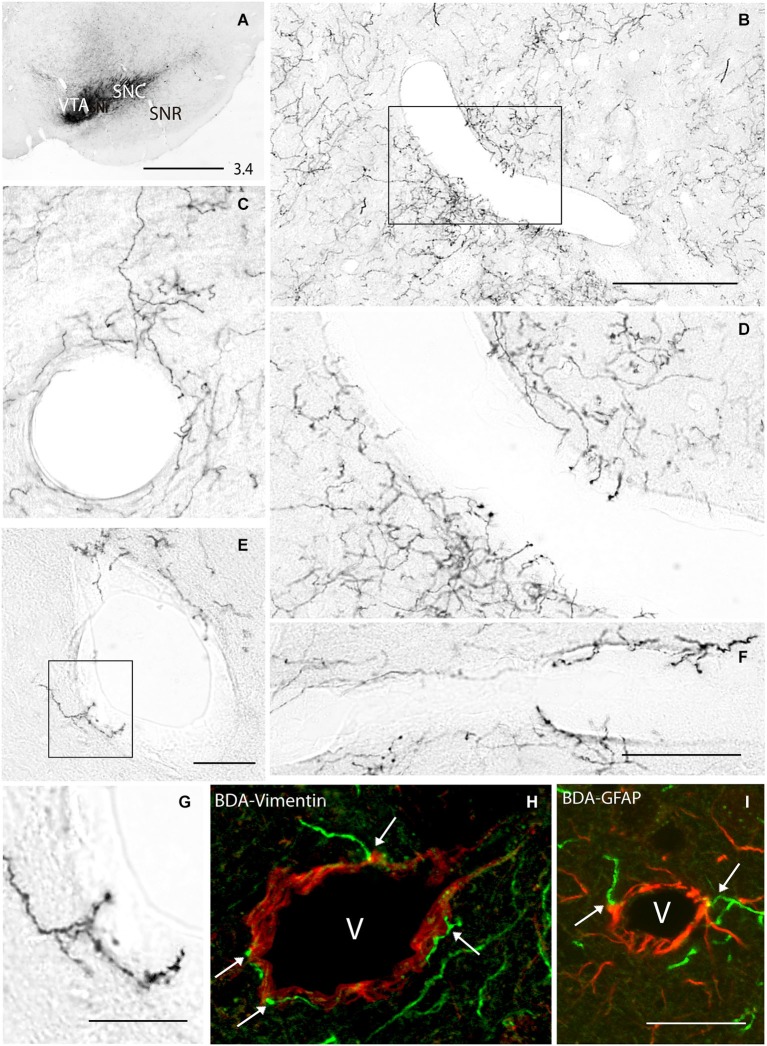
**(A)** Biotinylated dextran amine (BDA) injection in the substantia nigra pars compacta (SNC) and ventral tegmental area (VTA) of the rat. **(B–G)** BDA-positive fibers emitting terminals in close apposition to striatal vessels, some of them in the form of individual axons **(C,E,G)**, and others forming sparse plexuses **(B,D,F)**. **(H,I)** Double labeling for BDA and vimentin **(H)** or GFAP **(I)** showing terminals abutted on striatal vessels and surrounding astrocytes (arrows in **H** and **I**). **(D,G)** Boxed areas in **(B)** and **(E)** respectively. The number at the bottom right in **(A)** indicates the distance from the interaural axis in millimeters. SNR, substantia nigra pars reticulata. v, vessel lumen. Bar in **(A)**, 1 mm; in B, 400 μm; in **(F)** (for **D and F**), 150 μm; in **(G)**, 100 μm; in **(I)** (for **H and I**), 100 μm.

Striatal terminals were studied in rats whose BDA injection was restricted to the midbrain dopaminergic cell groups (substantia nigra pars compacta (SNC) and ventral tegmental area (VTA), Figure 1A). Cases with injection involving neighboring centers were excluded. Consistent with previous reports (Gerfen et al., 1987; Joel and Weiner, 2000), the regional distribution of striatal BDA positive fibers varied with the location of the injection site. Injections in VTA and/or the rostromedial region of the SNC provided a terminal field preferentially localized in the ventral striatum and the ventral part of the dorsal striatum, while after injections in the caudolateral region of the SNC, terminals spread out across the dorsal striatum.

Axons through the ventral and dorsal striatum showed collateral fibers emitting terminals in close apposition to the wall of blood vessels of diverse diameters (range 50–500 μm). They usually arise in the form of individual branches that emit isolated perivascular endings (Figures 1C,E,G), but sparse perivascular terminal plexuses were also found (Figures 1B,D,F). Double labeling for BDA and vimentin (a marker of intermediate filaments present in vessels and astrocytes) or GFAP (an astroglial marker) confirmed the presence of BDA-positive terminal and “en passant” buttons abutted on the wall of striatal vessels (Figure 1H) and astroglial cells wrapping them (Figure 1I).

**Figure 2:**
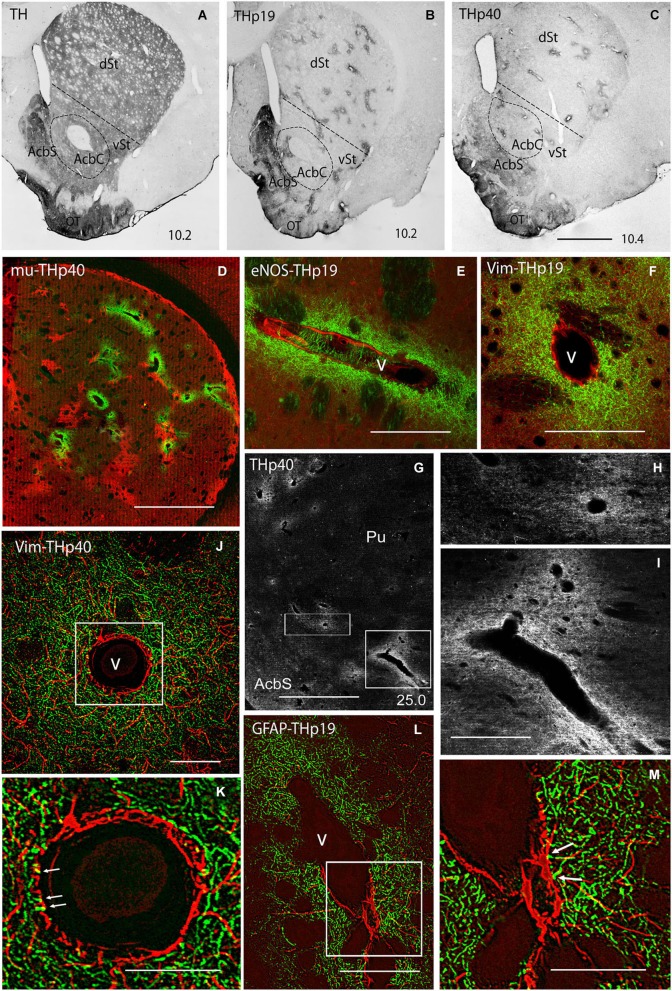
**(A–C)** Immunostaining for the non-phosphorylated (TH; **A**) and phosphorylated forms of tyrosine hydroxylase at Ser19 (THp19; **B**) and Ser40 (THp40; **C**) in rat striatum. We note that in contrast to the homogeneous labeling for TH **(A)**, THp19 and THp40 immunostaining is restricted to the olfactory tubercle (OT), accumbens shell (AcbS), and discrete regions throughout the ventral (vSt) and dorsal (dSt) striatum. **(D)** Double labeling for mu-opioid receptor (mu, a marker of striosomes; red) and THp40 (green) showing that THp40 is not localized in striosomes. **(E,F)** Double labeling for endothelial nitric oxide synthase (eNOS; **E**, red) or vimentin (Vim; **F**, red) and THp19 (green) showing dense plexuses of THp19-positive terminals surrounding longitudinal- **(E)** and transversally **(F)** cut vessels. **(G)** Immunostaining for THp40 in the monkey striatum showing intense immunoreactivity in perivascular terminals. **(H,I)** Boxed areas in **(G)**. **(J)** Double labeling for vimentin (Vim; red) and THp40 (green) in rat striatum subjected to deconvolution processing. **(K)** Boxed area in **(J)** showing THp40 terminals in close apposition to or penetrating (arrows) the vessel wall (arrows). **(L)** Double labeling for GFAP (red) and THp19 (green) in rat striatum subjected to deconvolution processing. **(M)** Boxed area in **(L)** showing THp19 terminals touching astroglial processes (arrows). Doted line in **(A–C)** indicates the putatitve border between vSt and dSt. The number at the bottom right in **(A–C,G)** indicates the distance from the interaural axis in millimeters. AcbC, accumbens core; Pu, putamen; v, vessel lumen. Bar in **(C)** (for **A–C**), 450 μm; in **(D)**, 200 μm; in **(E)**, 75 μm; in **(F)**, 75 μm; in **(G)**, 3 mm; in **(I)** (for **H and I**), 1 mm; in **(J)**, 50 μm; in **(K)**, 20 μm; in **(L)**, 50 μm; in **(M)**, 20 μm.

In order to confirm the dopaminergic nature of this projection, striatal sections of BDA-injected rats were further processed for TH (the rate-limiting enzyme in DA synthesis) and DAT. For TH immunohistochemistry, antibodies against the native (non-phosphorylated) as well as the phosphorylated forms of TH at serine 19 (THp19), serine 31 (THp31) and serine 40 (THp40) were used. Immunochemistry for native TH resulted in a diffuse striatal staining (Figure 2A), making it difficult to identify double TH-BDA positive axons. Consistent with previous reports supporting the idea that under basal conditions only a very small proportion of TH is phosphorylated (Haycock et al., 1998; Salvatore et al., 2001; Bobrovskaya et al., 2004), immunostaining for the phosphoryated TH (THp) forms was significantly lower than for native TH, and was restricted to discrete striatal regions. Thus, immunoreactivity for THp31 was virtually undetected in our material (data not shown), and for THp19 and THp40 was confined to the olfactory tubercle (OT), the accumbens shell and small patches throughout the ventral and dorsal striatum (Figures 2B,C). The similarity in shape and distribution between THp patches and striosomes (see Graybiel and Ragsdale, 1978) prompted us to perform double labeling for THp19 or THp40 and mu-opioid receptor (a marker of striosomes; Desban et al., 1993). However, no colocalization between both markers was found (Figure 2D), indicating that THp-rich patches preferentially localize in the matriceal compartment of the striatum. Interestingly, all the patches surround vascular profiles. The combination of immunolabeling for THp (Ser19 or Ser40) and two vessel markers, the endothelial form of nitric oxide synthase (eNOS, Figure 2E) and vimentin (Figure 2F), confirmed that THp-rich patches form dense terminal plexuses around striatal vessels, even penetrating their external layers (Figures 2J,K arrows). The presence of THp perivascular plexuses was also explored in monkeys. As shown in Figures 2G–I, small and large vessels in the ventral and dorsal striatum of monkeys were also surrounded by dense plexuses of THp terminals. Furthermore, double labeling for THp (Ser19 or Ser40) and GFAP in rats showed that THp-terminals are in close apposition to perivascular astroglial processes (Figures 2L,M arrows).

**Figure 3:**
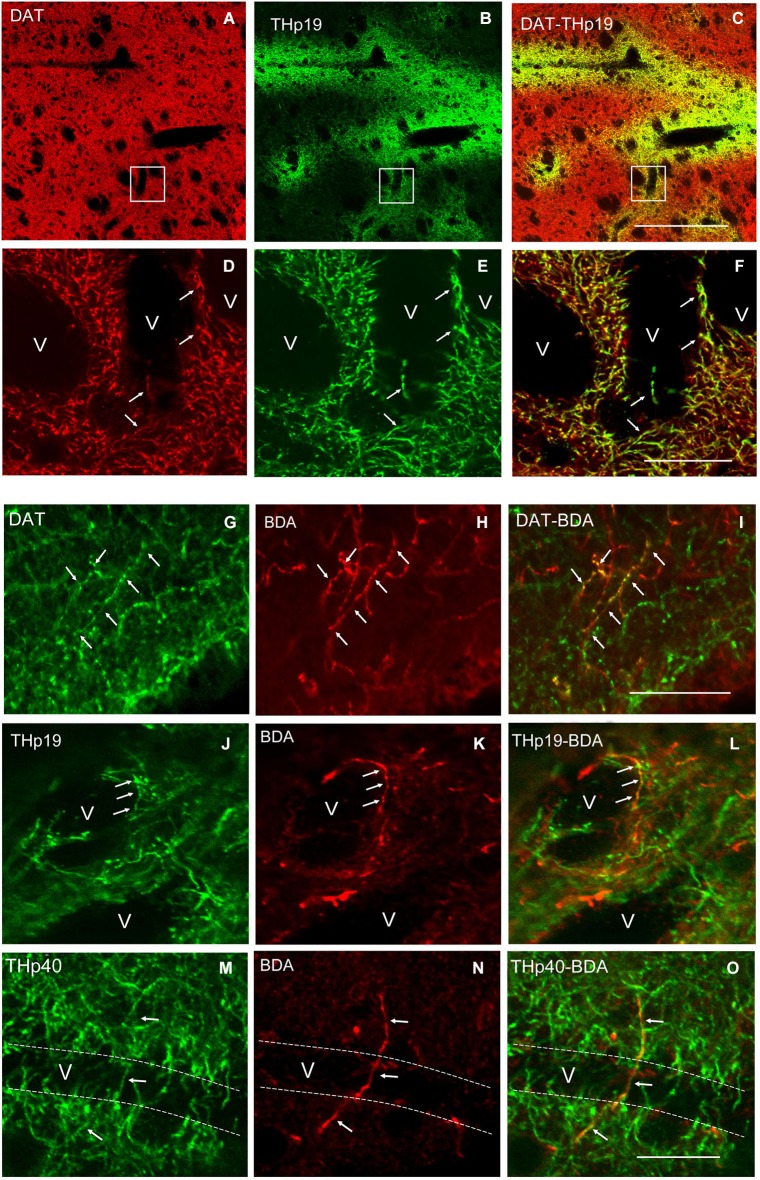
**(A–C)** A low magnification view of double labeling for the dopamine transporter (DAT) and THp19 in the dorsal striatum of the rat. **(D–F)** Boxed areas in **(A)**, **(B)** and **(C)** respectively showing that all perivascular THp19 fibers and terminals are dopaminergic (DAT-immunoreactive). **(G–O)** Double labeling for DAT, THp19 or THp40 and BDA showing that BDA-positive ascending axons **(H)** and perivascular terminals **(K,N)** are immunoreactive for DAT **(G–I)**, THp19 **(J–L)** and THp40 **(M–O)**. v, vessel lumen. Dotted lines in **(M–O)** indicate the probable localization of the vessel wall. Bar in **(C)** (for **A–C**), 130 μm; in **(F)** (for **D–F**), 40 μm; in **(I)** (for **G–I**), 40 μm; in **(O)** (for **J–O**), 40 μm.

**Figure 4:**
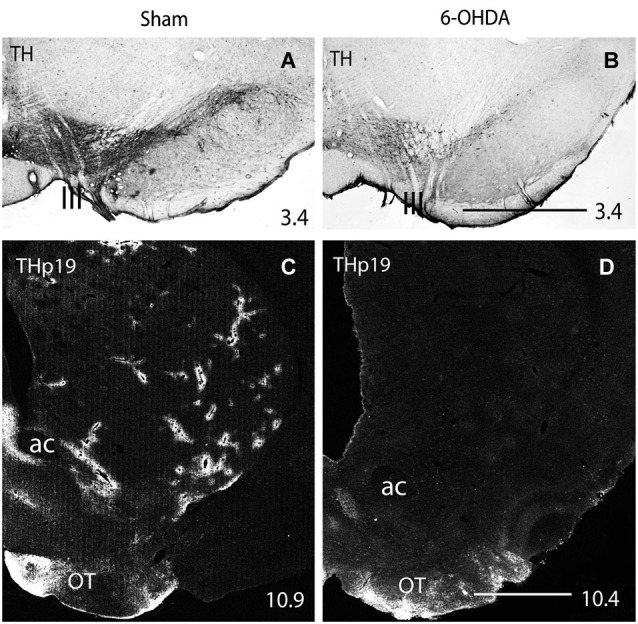
**(A,B)** Immunohistochemistry for TH in the rat midbrain after sham **(A)** and 400 μg 6-OHDA **(B)** intracerebroventricular injection. **(C,D)** Immunofluorescence for THp19 in the striatum of sham **(C)** and 400 μg 6-OHDA **(D)** injected rats. One can see the decrease in the number of TH-positive midbrain cells after 6-OHDA injection (compare **A** and **B**), and that THp-19 striatal perivascular plexuses disappear after midbrain DA-cell degeneration (compare **C** and **D**). ac, anterior commissure; OT, olfactory tubercle; III, fibers of the third cranial nerve emerging in the surface of the ventral midbrain. Numbers at the bottom right indicate the distance from the interaural axis in millimeters. Bar in **(B)** (for **A** and **B**), 1 mm; in **(D)** (for **C** and **D**), 1 mm.

As previously reported (González-Hernández et al., 2004), immunostaining for DAT showed a homogeneous field of well-defined striatal terminals (Figure 3A). A low magnification view of double labeling for DAT and THp (Ser 19 and Ser 40) in rats showed co-localization in practically all perivascular plexuses (Figures 3A–C). The count of double labeled terminals at higher magnification revealed that within 100 μm from striatal vessels, virtually 100% (98.4 ± 0.9%) of positive THp19 terminals were immunoreactive for DAT, and 97.7 ± 1.1% of DAT terminals were also immunoreactive for THp19 (Figures 3D–F). Furthermore, 97.4 ± 0.8% of THp40 terminals were immunoreactive for DAT, but THp40 immunoreactivity was not detected in 12.8 ± 1.3% of DAT terminals. These findings confirm the dopaminergic nature of THp plexuses and that both serine residues are phosphorylated in most perivascular terminals. Further supporting the midbrain dopaminergic origin of the THp-rich innervation of striatal vessels, BDA-positive axons and perivascular terminals were immunoreactive for both DAT (Figures 3G–I) and THp19 (Figures 3J–L) or THp40 (Figures 3M–O). Moreover, striatal THp perivascular plexuses disappeared after midbrain dopaminergic lesion in parallel with the loss of midbrain DA-cells (6-OHDA, Figure 4). In sum, our results indicate that striatal vessels receive midbrain dopaminergic inputs that form dense perivascular plexuses of THp-rich terminals.

## Discussion

The cerebral blood flow is controlled by two major neurovascular systems. One of them, known as the extrinsic system, consists of fibers from sympathetic and parasympathetic extracranial ganglia and the sensory trigeminal ganglion that release different vasoactive substances on the wall of extracerebral arteries and pial arterioles for the regulation of the global cerebral perfusion (Drake and Iadecola, 2007; Willie et al., 2014). The other one, known as the intrinsic system, consists of fibers arising from different neurons in the brain which project directly to parenchymal microvessels or astroglial end-feet adjoining them for the regulation of highly localized changes in blood flow coupled with regional synaptic activity (Iadecola, 2004; Hamel, 2006; Drake and Iadecola, 2007; Attwell et al., 2010; Lecrux and Hamel, 2011). This system, preferentially studied in the cerebral cortex, involves local GABAergic, peptidergic and glutamatergic collaterals, as well as cholinergic, noradrenergic and serotoninergic ascending inputs (Attwell and Iadecola, 2002; Cauli et al., 2004; Iadecola, 2004; Lecrux and Hamel, 2011). The use of anterograde tracers, specific neurotoxins and electron microscopy shows that these projections come from the nucleus basalis, locus coeruleus, and raphe nuclei respectively (Reinhard et al., 1979; Vaucher and Hamel, 1995; Cohen et al., 1997), and that their endings make contact with both the components of the neurovascular unit, the vessel wall and surrounding astrocytes (Vaucher and Hamel, 1995; Cohen et al., 1997). Dopaminergic neurotransmission has also been implicated in both neurovascular regulatory systems. Firstly, through *in vitro* and *in situ* studies showing that DA and its analogs promote vasoactive changes in convexity cerebral arteries and pial arterioles (Toda, 1976; Edvinsson et al., 1978a,b, 1985; Forster et al., 1983), and more recently, from the evidence of dopaminergic contacts in capillaries and perivascular astrocytes (Krimer et al., 1998), and the induction of hemodynamic changes in discrete brain regions by dopaminergic drugs (Marota et al., 2000; Choi et al., 2006; Sander et al., 2013) and their inhibition after dopaminergic lesion (Chen et al., 1997; Nguyen et al., 2000; Jenkins et al., 2004). However, the anatomical origin of dopaminergic inputs to intracerebral vessels has not been elucidated. The results here, based on the injection of an anterograde neuronal tracer, combination of vessel, astrocyte and dopaminergic markers, confocal microscopy and dopaminergic lesion, indicate that striatal vessels receive inputs from midbrain dopaminergic neurons. We are aware that only electron microscopy provides high enough magnification to properly identify synaptic structures. Nevertheless, high-resolution confocal laser scanning followed by adequate post-adquisition computer processing markedly reduces the resolution gap between optical and electron microscopies (Sibarita, 2005; Salin et al., 2008). Thus, one can suggest that, similar to dopaminergic and non-dopaminergic inputs to cortical vessels (Vaucher and Hamel, 1995; Cohen et al., 1997; Krimer et al., 1998), ascending dopaminergic terminals make contact with both components of the neurovascular unit in the rat striatum. In any case, we know that besides the synaptic transmission which requires interneuronal contacts, neurotransmitters and neuromodulators, including DA, can signal through extrasynaptic receptors after flowing short or long distances in the extracellular space. Interestingly, electron microscopy studies show that 50% of DA D_1_ and D_2_ receptors in the rat striatum are extrasynaptic (Yung et al., 1995), suggesting that most dopaminergic afferents can operate via both synaptic and extrasynaptic receptors. According to Fuxe et al. (2012), this sort of intercellular communication called volume transmission (Agnati et al., 1986), is the main communication pathway in neuroglial and neurovascular interactions. The evidence of DA D_1_ and D_2_ receptors in endothelial and astroglial cells (Bacic et al., 1991; Bal et al., 1994; Zanassi et al., 1999; Choi et al., 2006), together with the time (0–2 s) of vascular response to DA release in the striatum (Knutson and Gibbs, 2007) agree with the suggestion of Fuxe et al. (2012), and therefore the evidence of terminal contacts becomes less relevant.

Our results further show that dopaminergic axons innervating striatal vessels form dense perivascular plexuses immunoreactive for phosphorylated TH at Ser19 and Ser40. We know that phosphorylation is the primary mechanism responsible for short-term activation of TH, the rate-limiting enzyme of catecholamine synthesis, and that TH may be phosphorylated by different protein kinases at four serine residues (8, 19, 31 and 40) near N-terminus (Haycock and Wakade, 1992; Dunkley et al., 2004; Nakashima et al., 2009). However, under basal conditions no more than 5–7% TH is phosphorylated, and a substantial proportion of TH becomes phosphorylated only after protein kinase activating stimuli (Pocotte et al., 1986; Haycock, 1993; Haycock et al., 1998; Salvatore et al., 2001; Bobrovskaya et al., 2004). Consistent with these data, Xu et al. (1998) reported reduced immunoreactivity for phosphorylated TH in comparison with that for the native form, with phosphorylation being restricted to Ser19 and localized in the accumbens nucleus and the OT. Our results confirm these findings but enlarging them show that TH is also constitutively phosphorylated in dopaminergic terminals encircling vessels throughout the ventral and dorsal striatum. In addition, as demonstrated in rats and monkeys, phosphorylation also involves Ser40 residue.

The functional meaning and advantages of having multiple phosphorylatable positions have not been yet elucidated (for review see Daubner et al., 2011), but there is a general consensus in that Ser40 is critical for TH activity. Thus, while no activation mechanisms and functional consequences have been found for phosphorylation at Ser8 and phosphorylation at Ser19 and Ser31 promote no more than 2-fold increases in TH activity, phosphorylation at Ser40 results in a 300-fold decrease in TH affinity for catecholamines with a 20-fold increase in TH activity (Bevilaqua et al., 2001; Bobrovskaya et al., 2004; Dunkley et al., 2004; Daubner et al., 2011). In addition, although Ser19 phosphorylation “*per se*” does not significantly affect TH activity, it modifies TH structure thereby accelerating its phosphorylation at Ser40 (Bevilaqua et al., 2001). Consequently, one can assume that dopaminergic terminals with TH phosphorylated at Ser19 and Ser40 are responsible for the basal TH activity in the striatum, and that their DA content ready to be released is higher than in those without phosphorylated TH.

**Figure 5:**
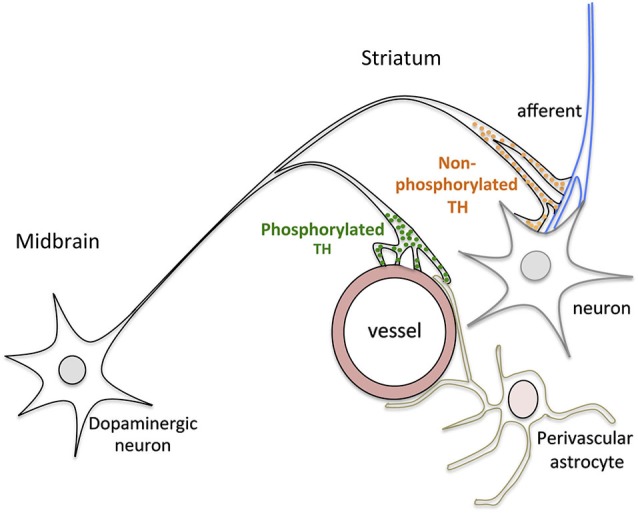
**Schematic drawing summarizing the main results of this study**. Dopaminergic terminals on vessels contain phosphorylated TH while those on striatal neurons and afferents contain non-phosphoryated TH, suggesting that DA release onto vessels precedes DA release onto neurons.

Studies about the striatal processing in associative learning indicate that the activity of striatal neurons in each trial is preceded by adaptive local hemodynamic changes which are mediated by DA (Knutson and Gibbs, 2007; Peterson and Seger, 2013). Midbrain dopaminergic neurons fire in response to reward-predicting cues leading to an increase in extracellular DA levels in the striatum (Roitman et al., 2004). Changes in extracellular DA levels are paralleled by an increase in the regional cerebral blood flow that is inhibited by dopaminergic lesion (Chen et al., 1997; Nguyen et al., 2000; Mandeville et al., 2001; Jenkins et al., 2004). Consequently, the prior release of DA in discrete striatal regions is nowadays considered a key factor in the regulation of blood flow depending on local metabolic demands. It is notable that dopaminergic signaling has also been involved in anticipatory hemodynamic changes in the cerebral cortex (Tan, 2009). However, in the light of our and previous results, this should be quite different to that in the striatum. In contrast to the conspicuous perivascular plexuses involving small to large vessels from the OT to the dorsal striatum, dopaminergic terminals are sparse and restricted to small vessels in the cerebral cortex (Krimer et al., 1998). Furthermore, as shown in rats and monkeys, TH is not constitutively phosphorylated in vessel terminals in the cerebral cortex. The restricted localization of phosphorylated TH in dopaminergic terminals of striatal vessels suggests that it is related to specific aspects of striatal processing. As shown in Figure 5, a striatal neurovascular unit and the neighboring neuropil probably receive projections from the same midbrain neuron. Consequently, dopaminergic inputs should reach both compartments at the same time. However, the fact that dopaminergic terminals in perivascular plexuses contain phosphorylated TH facilitates that the release of DA onto vessels precedes that onto the neighboring neuropil, and then, that DA vascular actions precede DA neuronal (non phosphorylated TH) actions.

We know that the striatum receives the most intense dopaminergic input in the brain, and that DA actions are exerted through different DA-receptors in striatal neurons and cortical and subcortical afferents arriving to different striatal regions (Obeso et al., 2008). In addition, the loss of DA signaling in striatal circuits is considered to cause motor symptoms as well as less noticeable cognitive and psychiatric manifestations of PD (Rodriguez-Oroz et al., 2009). However, as our results show, an important contingent of dopaminergic axons reaches striatal vessels rather than neurons, suggesting that these axons can play a role in the pathophysiology of PD. Beyond the concept of vascular parkinsonism (Kalra et al., 2010), the relationship between genuine PD and cerebral perfusion has mostly been focused on whether vascular co-morbidities can aggravate motor symptoms as a result of additive effects of two independent disorders (Kotagal et al., 2014), or the possibility that vascular aspects can act synergistically with other factors contributing to dopaminergic cell degeneration. In support of the latter, morphological changes have been found in nigral vessels of PD patients and animal models of PD (Faucheux et al., 1999; Barcia et al., 2005), and the density of nigral microvessels has also been found to be low in aged rats (Villar-Cheda et al., 2009). In addition, recent studies by Rodriguez-Perez et al. (2013) reveal that chronic brain hypoperfusion can “*per se*” induce dopaminergic cell degeneration and exacerbate the degeneration promoted by intrastriatal injection of 6-OHDA. Our findings suggest that independently of the vascular involvement in dopaminergic degeneration, the striatal blood flow can also be substantially affected as a result of dopaminergic cell degeneration. Consequently, the supply of oxygen and nutrients coupled to hemodynamic changes preceding neuronal activity might also be altered in the striatum, contributing as an additional factor in the pathophysiology of PD from its first stages. Interestingly, although the relationship between neuronal activity and oxygen consumption is the basis of functional imaging technologies widely used in the diagnosis of PD (Mahlknecht et al., 2010), nowadays blood flow is considered to merely serve as a metabolic support in this paradigm (Moore and Cao, 2008). Moreover, changes in striatal perfusion in the course of PD are still a matter of controversy. While some authors report an increase of the regional blood flow (Wolfson et al., 1985; Feigin et al., 2002; Hsu et al., 2007), others find no changes (Bissessur et al., 1997) or a decrease (Markus et al., 1994; Van Laere et al., 2004). These discrepancies reflect differences in data processing and the clinical profile of patients studied. In this respect, Imon et al. (1999) found a decrease in striatal blood flow at early stages of PD, and an increase at later stages, suggesting that changes can depend on the disease stage. Bearing in mind the relative weight of the “vascular component” in the mesostriatal pathway, it is possible that hemodynamic changes may be detected and monitored from the early stages of PD with the systematic use of functional imaging.

## Conflict of interest statement

The authors declare that the research was conducted in the absence of any commercial or financial relationships that could be construed as a potential conflict of interest.
